# Eosinophilic Gastrointestinal Disorder in Coeliac Disease: A Case Report and Review

**DOI:** 10.1155/2012/124275

**Published:** 2012-12-17

**Authors:** Dennis N. F. Lim, Annelise Wilkins, Laura Elizabeth Horton, Ishfaq Ahmad, Catherine lo Polito, Chris Phillips

**Affiliations:** Department of Gastroenterology, Alexandra Hospital, Worcestershire Acute Hospitals NHS Trust, Woodraw Drive, Redditch B98 7UB, UK

## Abstract

Eosinophilic gastrointestinal disorder is a rare disorder characterised by eosinophilic infiltration of the gastrointestinal tract. There are various gastrointestinal manifestations with eosinophilic ascites being the most unusual and rare presentation. Diagnosis requires high index of suspicion and exclusion of various disorders associated with peripheral eosinophilia. There are no previous case reports to suggest an association between eosinophilic gastrointestinal disorder and coeliac disease in adults. We report a case of eosinophilic ascites and gastroenteritis in a 30-year-old woman with a known history of coeliac disease who responded dramatically to a course of steroids.

## 1. Introduction

Eosinophilic gastrointestinal disorder is an unusual inflammatory gastrointestinal disease characterized by eosinophilic gut infiltration [1]. There have been case series and cohort studies suggesting an association between eosinophilic oesophagitis and coeliac disease in children. However, there were no previous reports or studies to indicate an association between coeliac disease and eosinophilic gastrointestinal disorder in adults. This paper examines a patient with concurrent eosinophilic gastrointestinal disorder and coeliac disease.

## 2. Case Report

**Figure 1:**
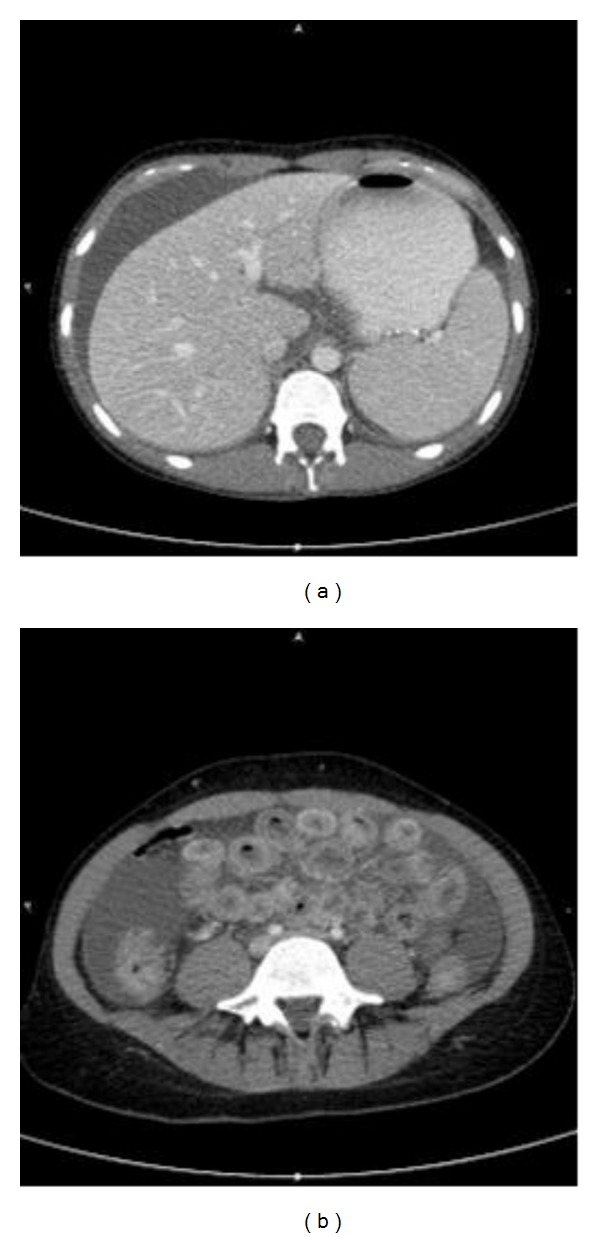
Computed tomography of abdomen and pelvis showing large amount of ascites and thickened small bowel wall. No pelvis or peritoneal mass identified.

**Figure 2:**
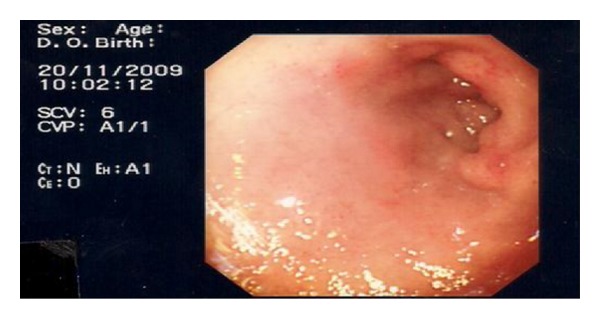
Esophagogastroduodenoscopy showed multiple areas of erythematous patches at the duodenal bulb.

**Figure 3:**
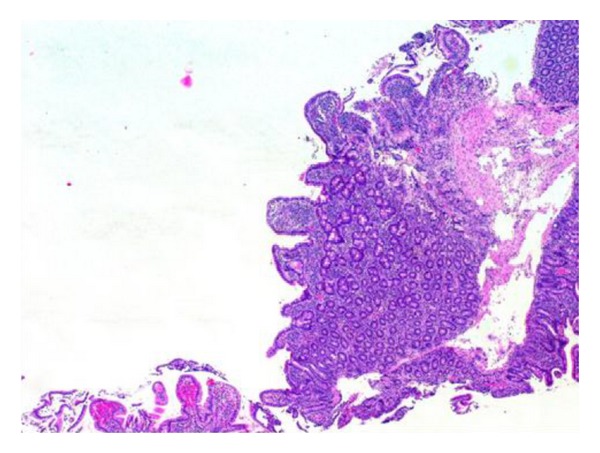
H&E Low power view of second part of duodenal biopsy showing Marsh 3a villous atrophy.

 A 30-year-old Caucasian female presented with a two-week history of central abdominal pain and distension, vomiting, and increased frequency of bowel motion. There had been no weight loss or history of foreign travel. She was known to have coeliac disease diagnosed at age two. She was compliant to gluten-free diet. There was no history of drug allergy, asthma or skin eczema. Abdominal examination showed sign of ascites. There were no stigmata of chronic liver disease. The rest of the systemic examination was also within normal limits. A summary of her laboratory investigations is presented in Table 1. Computed tomography of the abdomen and pelvis showed large volume ascites and prominent small bowel loops (Figures 1(a) and 1(b)). There was no evidence of a pelvic or peritoneal mass. A diagnostic paracentesis revealed straw-coloured ascites. Analysis of the ascitic fluid is summarized in Table 1. Oesophagogastroduodenoscopy (Figure 2) showed multiple areas of erythematous patches at the superior duodenal angle close to the second portion of the duodenum. Colonoscopy and biopsies of colonic and terminal ileal mucosa were normal. Histological examination of duodenal mucosa revealed Marsh 3a villous atrophy consistent with coeliac disease although no previous biopsies were available for comparison (Figure 3).

**Figure 4:**
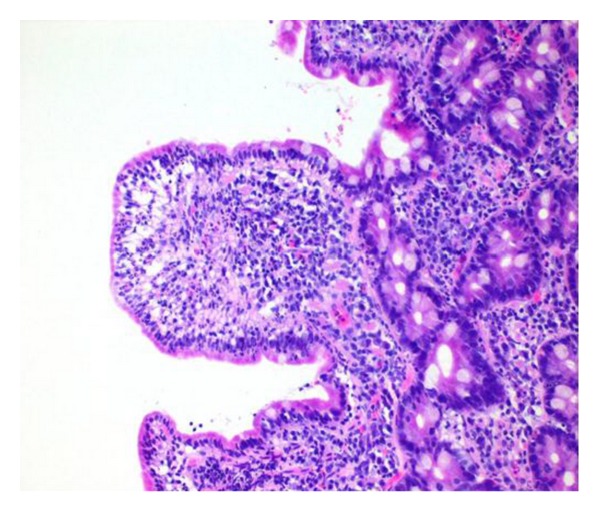
H&E high power view of jejunal biopsy showed increase in lamina propria cellularity with eosinophilic infiltration.

**Figure 5:**
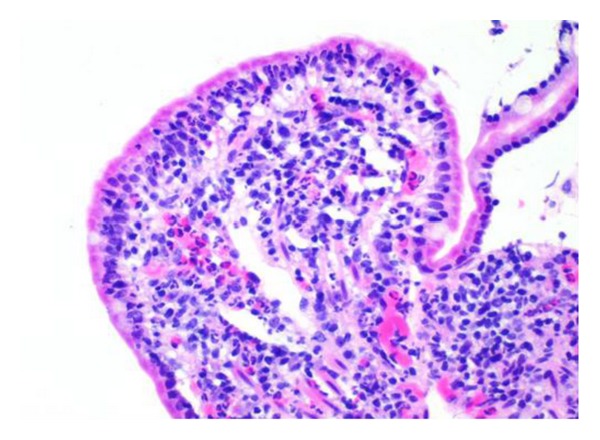
H&E section of jejunal biopsy showed eosinophils infiltration (30/HPF) in the lamina propria.

Jejunal mucosa showed dense infiltration of eosinophils on the lamina propria (30/HPF) (Figures 4 and 5). There was no histological evidence of an enteropathy-associated T-cell lymphoma, granuloma, or malignancy. Unfortunately, the endoscopist did not obtain oesophageal and gastric biopsies. 

 Laporoscopic examination showed no evidence of underlying intra-abdominal malignancy. After excluding the possibilities of malignancy, parasitic disease and an autoimmune disorder, eosinophilic gastrointestinal disorder was diagnosed. This diagnosis was based on the presence of peripheral eosinophilia, eosinophilic ascites, and eosinophilic infiltration of the proximal duodenum and jejunal mucosa. 

 The patient was started on intravenous hydrocortisone 100 mg four times a day with rapid resolution of her symptoms and eosinophilia. She received three days of intravenous hydrocortisone and was converted to a reducing dose of oral prednisolone 40 mg per day for four days. This was reduced to 20 mg per day for four days, 10 mg per day for three days, 5 mg per day for five days, then 2.5 mg per day for seven days, and stop. 

**Figure 6:**
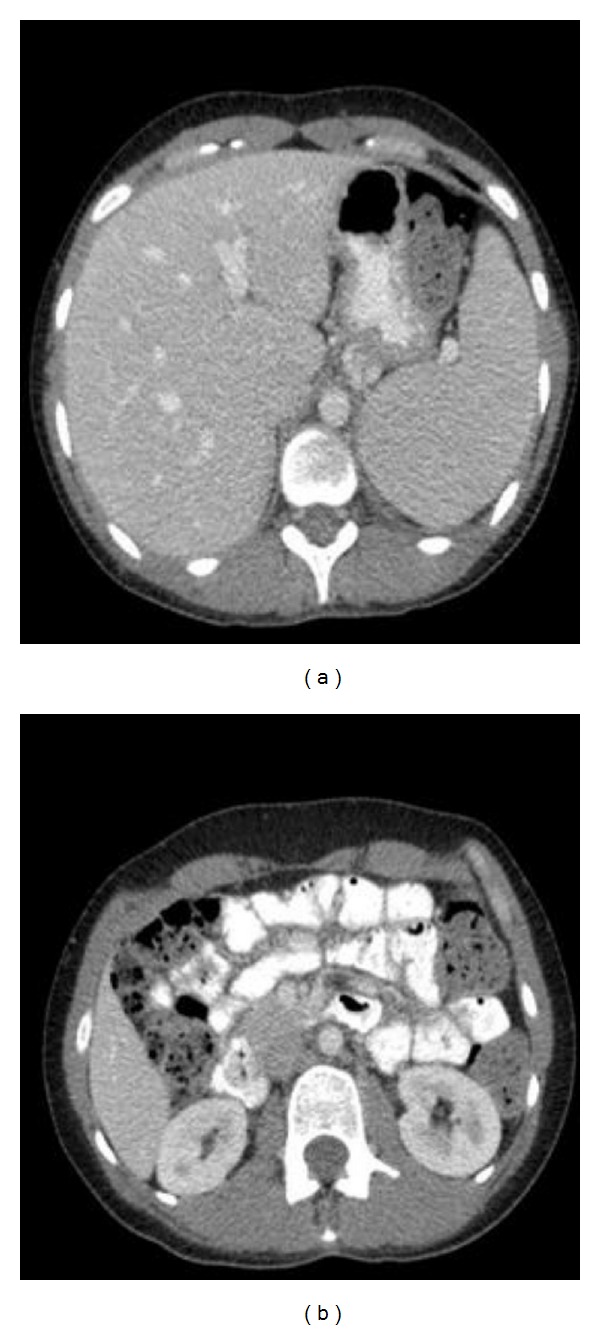
Computed tomography showing resolution of ascites and small bowel thickening three months after discharge from hospital.

However, the length of steroid treatment was greatly influenced by the development of steroid-induced psychosis. The patient was eventually admitted for psychiatric treatment following discharge. Her psychosis had resolved upon discontinuation of steroid and did not require long-term antipsychotic treatment. A repeat computed tomography of the abdomen and pelvis three months after discharge from hospital showed resolution of the ascites and small bowel wall thickening (Figures 6(a) and 6(b)). 

## 3. Discussion

 Eosinophilic gastrointestinal disorder is a rare disorder first described in 1937 [2]. The disorder is classified according to the predominance of eosinophilic infiltration in different layers of the intestinal wall: the mucosa, muscle, and subserosal layers [1]. Depending upon the area involved, the disease can present as eosinophilic oesophagitis, eosinophilic gastroenteritis, eosinophilic ascites, and eosinophilic colitis. 

 In a retrospective study of 40 patients, the most common symptoms were abdominal pain, nausea, vomiting, and diarrhoea. In this study, the percentages involving mucosal layer, muscle, and subserosal layers were 58%, 30%, and 12% respectively [3]. 

 Radiological features of eosinophilic gastroenteritis include thickening of mucosal folds on barium study or computed tomography depending on the severity and layer(s) involved [4]. However, similar changes may also be seen in Menetrier's disease, lymphoma, and Crohn's and granulomatous disease. Therefore, the thickening of bowel wall is not specific for eosinophilic gastroenteritis. The endoscopic appearance in eosinophilic gastroenteritis is also nonspecific, including erythematous, friable, nodular, and occasional ulcerative changes [5]. Mucosal biopsies are nondiagnostic in approximately 10% of cases either because of sampling error or patchy involvement. Therefore, a minimum of eight biopsies is recommended to establish the diagnosis [3, 6, 7]. 

Both cellular and humoral immunity have been postulated placing eosinophilic gastroenteritis between purely IgE-mediated disorders such as food allergies and non-IgE-mediated disorders such as inflammatory bowel disease and coeliac disease [8, 9]. It is known that activated eosinophils are found within the lamina propria of patients with coeliac disease, especially in the later stages after lymphocyte activation. These eosinophils have the capacity to synthesise IL-5, a cytokine produced by T and mast cells, which is the major actor involved in eosinophils differentiation and activation [8]. Although a subset of these patients has an allergic etiology and mucosal disease; there is a good deal of heterogeneity in the disease in regard to both allergic sensitization and anatomic location. Histopathological examination of the biopsies and ascetic fluid analysis are essential parts of the diagnostic workup. 

 The patient in our case report has a childhood history of coeliac disease at the age of two from another hospital. The diagnosis was based on gastrointestinal symptoms of ascites and gastroenteritis, the presence of dense eosinophil infiltration on the lamina propria of duodenal and jejunal biopsies, marked eosinophilia in the ascites, and the rapid response to steroid therapy [3]. However, unlike eosinophilic oesophagitis, there is no consensus definition of eosinophilic gastroenteritis or well-accepted cutoff eosinophil number per high power field. A large proportions of patients diagnosed with eosinophilic gastroenteritis responded to oral steroid in a dramatic fashion [1, 3]. The appropriate duration of steroid treatment remains unknown but improvement usually occurs within two weeks regardless of the layer of bowel involved [10]. Some patients may require prolonged therapy to induce resolution of symptoms [10]. In a small proportion of patients with a relapsing-remitting course, long-term low-dose steroids or immunosuppressive therapy may be required. A novel approach with the use of leukotriene receptor antagonists (Montelukast) has been successfully used in treatment of serosal eosinophilic gastroenteritis as steroid-sparing therapy [11]. This treatment is important especially in the paediatric patients. In addition, the use of monoclonal antibodies against IgE and IL5 such as omalizumab and mepolizumab, respectively, has shown promising results in the treatment of patients with eosinophilic gastroenteritis in clinical trials [9, 12].

## 4. Conclusions

 Elevated eosinophils in blood and different tissues have been found in coeliac disease. The coexistences of eosinophilic gastrointestinal disorder and coeliac disease in an adult should be considered when a patient presented with ascites in the absence of liver disease and unexplained gastrointestinal symptoms. 

## Figures and Tables

**Table 1:** Summary of laboratory results.

Peripheral eosinophil count	860 IU/L (normal 12–760 IU/L)
IgE	98 IU/L (normal 13–128 IU/L)
Tissue transglutaminase IgA	Negative 36.4 IU/L (normal = negative)
Tissue transglutamase antibody IgG	Negative (normal = negative)
HIV, thyroid function	Negative
Stool	Negative for oval, cysts and parasites
Parasites serologies (Echinococcus, Entamoeba histolytica, Toxocara, Trichinella)	Negative
RAST (radioallergosorbent test) egg, peanut, soya, milk, cheese, wheat, cod, mussel, salmon, shrimp, tuna, hazel, Brazil, and almond nuts	Negative tests for allergens
Ascitic fluid analysis	
Total protein	4.5 g/dL
Albumin	2.4 g/dL
SAAG (serum ascitic-albumin gradient)	0.1 g/dL
Eosinophil count	6030/mm^3^
Cytology	No evidence of malignant or lymphoma cells
